# Novel 3D Capacitors: Integrating Porous Nickel-Structured and Through-Glass-Via-Fabricated Capacitors

**DOI:** 10.3390/nano15110819

**Published:** 2025-05-28

**Authors:** Baichuan Zhang, Libin Gao, Hongwei Chen, Jihua Zhang

**Affiliations:** State Key Laboratory of Electronic Thin Films and Integrated Devices, School of Integrated Circuit Science and Engineering, University of Electronic Science and Technology of China, Chengdu 611731, China; zabooish@outlook.com (B.Z.); hwchen@uestc.edu.cn (H.C.); jhzhang@uestc.edu.cn (J.Z.)

**Keywords:** 3D capacitors, nanoporous structure, capacitance density, through-glass via, interdigitated blind-via array, withstand voltage, energy conversion efficiency

## Abstract

In this research work, two distinct types of three-dimensional (3D) capacitors were successfully fabricated, each with its own unique features and advantages. The first type of capacitor is centered around a 3D nanoporous structure. This structure is formed on a nickel substrate through anodic oxidation. After undergoing high-temperature thermal oxidation, a monolithic Ni-NiO-Pt metal–insulator–metal (MIM) capacitor with a nanoporous dielectric architecture is achieved. Structurally, this innovative design brings about several remarkable benefits. Due to the nanoporous structure, it has a significantly increased surface area, which can effectively store more charges. As a result, it exhibits an equivalent capacitance density of 69.95 nF/cm^2^, which is approximately 18 times higher than that of its planar, non-porous counterpart. This high capacitance density enables it to store more electrical energy in a given volume, making it highly suitable for applications where miniaturization and high energy storage in a small space is crucial. The second type of capacitor makes use of Through-Glass Via (TGV) technology. This technology is employed to create an interdigitated blind-via array within a glass substrate, attaining an impressively high aspect ratio of 22.5:1 (with a via diameter of 20 μm and a depth of 450 μm). By integrating atomic layer deposition (ALD), a conformal interdigital electrode structure is realized. Glass, as a key material in this capacitor, has outstanding insulating properties. This characteristic endows the capacitor with a high breakdown field strength exceeding 8.2 MV/cm, corresponding to a withstand voltage of 5000 V. High breakdown field strength and withstand voltage mean that the capacitor can handle high-voltage applications without breaking down easily, which is essential for power-intensive systems like high-voltage power supplies and some high-power pulse-generating equipment. Moreover, due to the low-loss property of glass, the capacitor can achieve an energy conversion efficiency of up to 95%. Such a high energy conversion efficiency ensures that less energy is wasted during the charge–discharge process, which is highly beneficial for energy-saving applications and systems that require high-efficiency energy utilization.

## 1. Introduction

The continuous evolution of microelectronics fabrication technology has enabled remarkable enhancement in device integration density, particularly through the transition from micrometer-scale to nanoscale semiconductor processes. This progression has driven an exponential demand for passive electronic components (e.g., resistors, inductors, and capacitors) that operate without external power requirements. Concurrently, advancements in semiconductor manufacturing have facilitated dimensional scaling of devices, allowing for a threefold increase in component density per unit volume compared to conventional architectures. Conventional approaches employing PIP (Positive–Insulator–Positive) and MOS (metal-oxide semiconductor) capacitor configurations have encountered persistent challenges: (1) interfacial oxidation at dielectric/non-metallic electrode boundaries induces capacitance degradation, particularly in oxygen-rich environments; (2) inherent material limitations result in elevated dissipation factors (tanδ > 0.05) and substantial parasitic capacitance (>15% of nominal value), significantly compromising high-frequency performance.

In contrast to conventional dielectric capacitors, metal–insulator–metal (MIM) architectures demonstrate superior power density (typically > 100 mW/mm^2^) with exceptional pulse current handling capability (>10 A/mm^2^), making them ideal for burst-mode power delivery systems [1,2,3,4,5,6,7,8,9]. This non-Faradaic storage mechanism eliminates redox reactions at the electrode–electrolyte interface, achieving sub-nanosecond charge/discharge rates (τ < 500 ps) with Coulombic efficiency exceeding 99.98%. The metallic electrode configuration provides dual advantages: (1) ohmic contact resistance below 10 μΩ·cm^2^ at the dielectric interface, and (2) parasitic capacitance reduction to <5% of nominal value through optimized work function matching. These characteristics underpin their critical role in modern DRAM architectures [10,11,12,13] requiring 30% capacitance density enhancement per technology node, 5G RF front-end modules have high requirements for the quality factor at high frequencies [14,15,16], precision analog systems demonstrating <0.01% voltage coefficient variation, and high-speed decoupling networks [17] compliant with JEDEC Wide I/O 2 specifications. Despite the demonstrated advantages of MIM capacitors, two critical shortcomings persist in state-of-the-art devices. First, planar thin-film electrodes offer only limited interfacial area, capping achievable capacitance density and exacerbating dielectric-loss effects at high frequencies. Second, many high-κ dielectrics (e.g., HfO_2_, Ta_2_O_5_) undergo interfacial reactions and early breakdown when subjected to strong electric fields, and their complex deposition routes drive up fabrication costs. Thus, there remains a clear need for three-dimensional architectures that can both multiply electrode–dielectric contact area by orders of magnitude, ensure high breakdown voltage under aggressive electrical conditions, and reduce fabrication complexity. Nickel, as an important engineering material, has been widely utilized in various fields such as batteries, fuel cells, supercapacitors, and catalytic processes in the chemical industry. In these applications, the factors governing chemical reactions are closely related to the specific surface area of metallic nickel. Therefore, developing porous nickel structures with high specific surface area has become a research hotspot in recent years. Porous nickel offers advantages such as low density, excellent energy absorption, permeability, and electromagnetic shielding capabilities. Moreover, nickel is cost-effective and environmentally friendly, further supporting its broad application prospects. The integration of porous metals with MIM (metal–insulator–metal) capacitor structures to fabricate high-surface-area capacitors has emerged as a significant focus in current research. TGV (Through-Glass Via) technology has emerged as a core enabling technique for advanced packaging and heterogeneous 3D integration, owing to its excellent dielectric properties, stable high-frequency signal transmission, and three-dimensional integration capability. Compared with Through-Silicon Vias (TSVs) and organic substrates, glass offers inherently low dielectric loss and high insulation strength, making it an ideal platform for the design and fabrication of high-density 3D capacitors. However, existing research has predominantly focused on the interconnection applications of TGVs, and their potential in energy storage devices remains largely underexplored.

In this paper, to increase the electrode contact area of capacitors in order to enhance capacitance density and reduce loss, this work fabricated a three-dimensional porous structure for the dielectric layer using anodic oxidation. This work analyzed the effects of different current densities during the anodization process on the formation of the porous structure and the subsequent impact on the capacitor’s capacitance density. Additionally, this work investigated the influence of various high-temperature oxidation times on the capacitance density during the preparation of the nickel oxide thin film. After determining the optimal current density and high-temperature oxidation duration, this work employed a magnetron sputtering process to deposit the top electrode onto the nickel oxide thin film, thus forming a three-dimensional porous Ni-NiO-Pt monolayer dielectric MIM capacitor. Testing results showed that the equivalent capacitance density reached 69.95 nF/cm^2^, which is 18 times higher than that of a planar non-porous nickel oxide capacitor. To enhance the breakdown voltage of capacitors in order to accommodate high-voltage pulsed systems. A blind-via array with a double-sided interdigitated geometry was fabricated in photosensitive glass via the laser-induced wet etching (LIWE) process. The evolution of the via structures was characterized under different laser focal positions. During metallization, both magnetron sputtering and atomic layer deposition (ALD) were employed; comparative analysis revealed that ALD achieved superior filling uniformity. Electrical measurements showed an equivalent capacitance density of 49.6 nF/cm^2^—approximately a 439-fold increase over planar-glass capacitors—while the breakdown field reached 8.2 MV/cm (corresponding to a 5000 V withstand voltage). An energy conversion efficiency of 95% was also attained.

## 2. Materials and Methods

### 2.1. Porous Nickel Oxide MIM Capacitor

**Figure 1:**
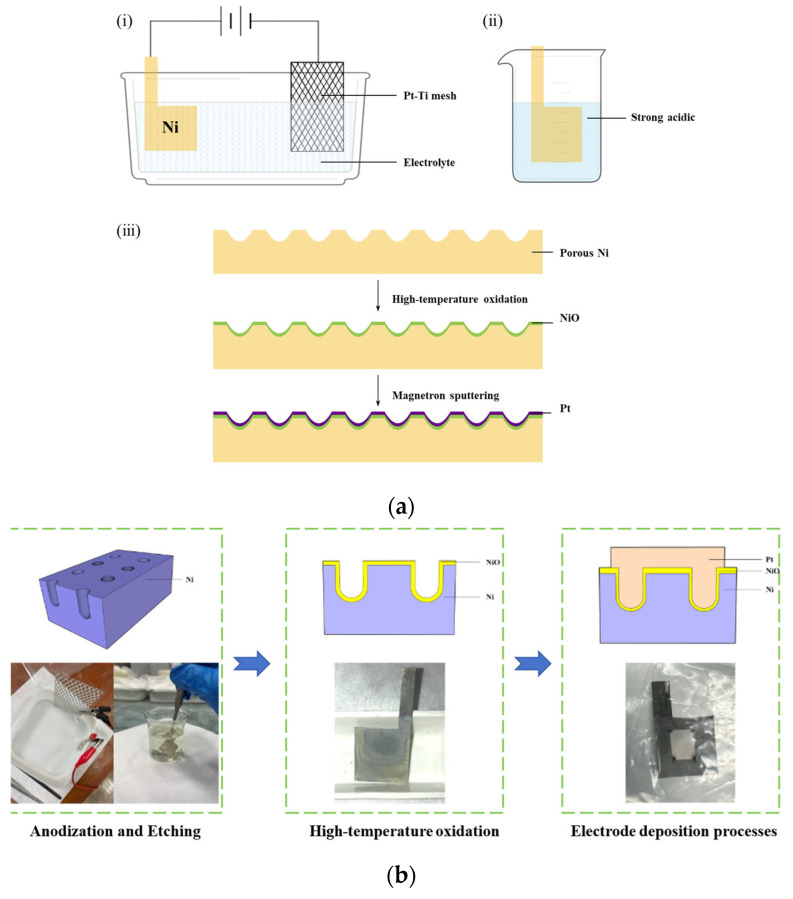
Fabrication process and structural schematics of the 3D porous NiO capacitor: (**a**) (**i**) anodization; (**ii**) etching; (**iii**) high-temperature oxidation, and electrode deposition processes; (**b**) schematic of the porous structure and cross-sectional view of the capacitor and experimental procedure and physical images.

Figure 1 shows the preparation process of a porous nickel oxide capacitor. The fabrication process commenced with anodic oxidation of nickel foils (thickness: 0.05 mm; purity: 99.99%) in a precisely formulated electrolyte containing 0.2 M deionized water (deionized water, NH_4_F and ethylene glycol sourced from Chengdu KE SHI Chemical Co., Ltd., Chengdu, China), 0.02 M NH_4_F, and ethylene glycol, maintained at 25 °C under a constant current density of 20 mA/cm^2^ for 20 min using a Keithley 2400 Source Meter, with concurrent magnetic stirring at 800 rpm to mitigate bubble formation at the electrode interface. The surface treatment procedure involved immersion in a laboratory-formulated ternary acid solution (H_3_PO_4_/HNO_3_/H_2_SO_4_ = 3:1:1 by volume), created by mixing individual acid components (all reagents purchased from Chengdu Kelong Chemical Co., Ltd., Chengdu, China) to achieve synergistic contaminant removal capabilities. This 5 min chemical etching protocol successfully eliminated surface impurities while maintaining structural integrity, resulting in a porous nickel substrate. Thermal treatment in a horizontal tube furnace involved controlled oxidation at 400 °C under oxygen flow (50 sccm, purity 99.999%) to form Ni_2_O_3_, followed by annealing at 600 °C in forming gas (95% N_2_/5% H_2_, 100 sccm total flow) to achieve stoichiometric NiO with cubic crystalline structure. The Pt layer was deposited using a magnetron sputtering system (JGP560, Shenyang Scientific Instrument Co., Ltd., Shenyang, China). First, the chamber was evacuated by starting the mechanical pump and molecular pump after sealing the chamber. When the base pressure reached 5 × 10^−4^ Pa, high-purity Ar gas (99.999%, 30 sccm) was introduced until the working pressure stabilized at 0.3 Pa. A DC sputtering power of 250 W was applied with a substrate rotation speed of 5 rad/min at room temperature for 5 min. The thickness of the Pt layer, measured by a Dektak 150 profilometer, was 200 nm. The resistivity of the Pt layer was measured using a four-point probe method. The four probes were arranged equidistantly, with the outer two probes connected to a constant current source to apply current and the inner two probes linked to a voltmeter to measure the voltage. Voltage and current measurements were separated to minimize the influence of contact resistance. The sheet resistance of the Pt layer was measured to be less than 1 Ω/□.

### 2.2. Glass-Based Three-Dimensional Capacitors

**Figure 2:**
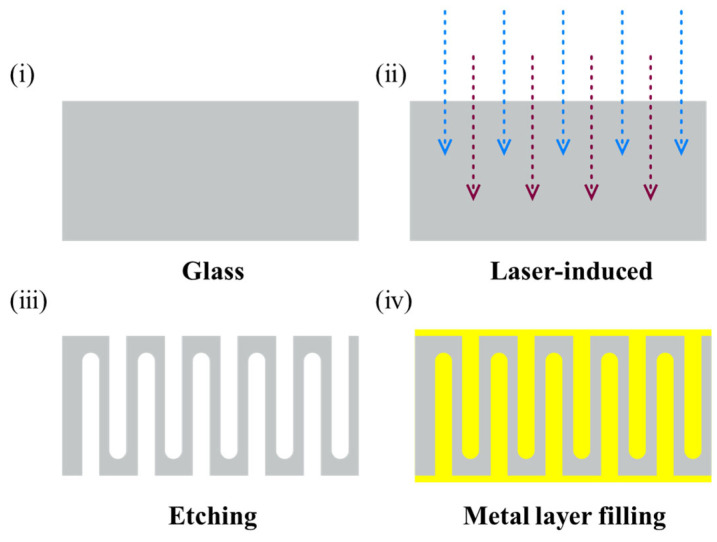
Process sequence for the fabrication of glass-based three-dimensional capacitors. (**i**) Glass substrate; (**ii**) Laser-induced; (**iii**) Etching; (**iv**) Metal layer filling.

Photosensitive glass blocks were processed into substrates via sequential cutting, grinding, and polishing steps. Sintered glass blocks were first diced into 2-inch square pieces with a final thickness of 1 mm using a diamond-wire saw operating at a feed rate of 0.3 mm/min. The as-cut samples were then thinned by double-side grinding, which ensures superior planarity on both faces compared to single-side grinding. By optimizing grinding pressures (both down-force and back-force) and wheel rotational speed, the substrates were reduced to a thickness of 700 µm. Subsequently, the 700 µm-thick specimens underwent chemical-mechanical polishing on a four-zone pressure polisher using a ceria slurry modified with sodium citrate to adjust the pH. The polishing mechanism relies on glass surface hydrolysis to form a silica gel layer, which is mechanically abraded to achieve further thinning. Through iterative polishing trials, the substrates were uniformly thinned to 500 µm. The polished 2-inch, 500 µm-thick glass substrates were then subjected to laser modification using a picosecond laser system (SLCU-11075I, Shengxiong Co., Ltd., Dongguan, China). Following laser exposure, the substrates were etched in 30 wt% HF solution (Chengdu Kelong Co., Ltd., Chengdu, China) for 30 min to produce the interdigitated blind-via array. Finally, electrode layers were deposited by either magnetron sputtering or atomic layer deposition (ALD) to form three-dimensional interdigitated capacitors. The overall fabrication workflow is illustrated in Figure 2.

Microstructural characterization was performed using a ZEISS Sigma 300 FE-SEM to analyze current density effects (10–40 mA/cm^2^) on nanopore morphology evolution. Capacitance–voltage (C-V) characterization using an Agilent 4294A precision impedance analyzer enabled equivalent capacitance density derivation from C-V hysteresis measurements. The I-V curves of the capacitor were measured using an Agilent 4156C semiconductor parameter analyzer to calculate the leakage current density. The voltage sweep range was set from −3 V to 3 V with a linear scan at a step size of 0.1 V. The current compliance was set slightly higher than the anticipated maximum current to prevent overcurrent damage to the sample. The NPLC (Number of Power Line Cycles) was configured to 1 to improve the signal-to-noise ratio, and a dwell time of 100 ms per voltage step was applied to ensure current stabilization. Energy charge–discharge properties of the capacitor were measured using an RC circuit. The charge–discharge experiment was conducted with a charge–discharge system (Agilent B1505A, Santa Clara, CA, USA) equipped with a high-voltage metal-oxide semiconductor field-effect transistor (MOSFET) switch (BehlkeHTS81). The 3D interdigital electrodes capacitor was charged by applying an electric field through the high-voltage MOSFET switch. Subsequently, the stored energy was discharged to two resistors (RL = 20 MΩ). The impedance Z was measured in the frequency range from 200 Hz to 1 MHz at room temperature with a precision LCR Meter (6500P, Wayue Kerr, London, UK).

## 3. Results and Discussion

### 3.1. Morphological Characterization and Mechanism Analysis: Porous Nickel Oxide MIM Capacitor

**Figure 3:**
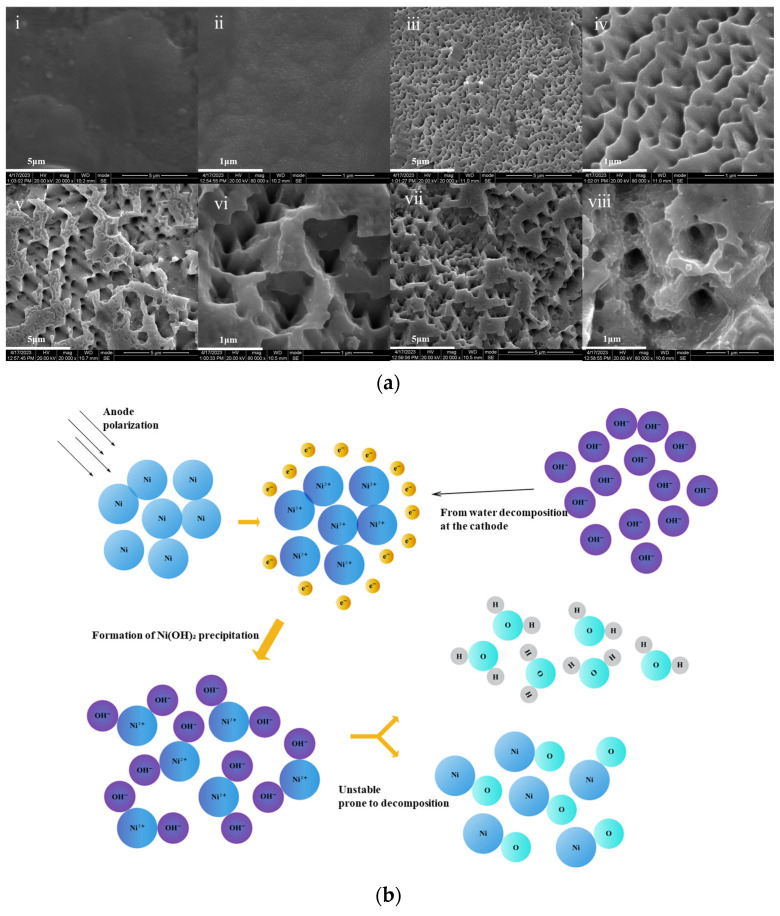
(**a**) Porous nickel morphologies under different current densities (**i**,**ii**) 10 mA/cm^2^, (**iii**,**iv**) 20 mA/cm^2^, (**v**,**vi**) 30 mA/cm^2^, (**vii**,**viii**) 40 mA/cm^2^; (**b**) anodic oxidation mechanism of Ni.

Figure 3a presents 20,000× SEM micrographs of porous nickel surfaces fabricated under current densities of 10–40 mA/cm^2^, with insets showing 80,000× magnification. At 10 mA/cm^2^ (Figure 3a(i,ii)) the surface exhibited underdeveloped porosity with limited specific surface area enhancement. Optimal pore formation occurred at 20 mA/cm^2^ (Figure 3a(iii,iv)), demonstrating uniform honeycomb-like architecture where higher magnification revealed coherent pore walls and minimized structural defects. Current densities exceeding 20 mA/cm^2^ induced progressive degradation: 30 mA/cm^2^ (Figure 3a(v,vi)) caused localized pore wall collapse, while 40 mA/cm^2^ (Figure 3a(vii,viii)) led to complete network dissolution with a 52% reduction in effective surface area compared to 20 mA/cm^2^ samples. These results quantitatively establish 20 mA/cm^2^ as the critical current density for achieving high-quality porous dielectric layers. By comparing the capacitance density of planar NiO dielectric capacitors and porous NiO capacitors with identical dielectric layer thicknesses, it can be calculated that the electrode area exhibits an 18-fold enhancement.

**Figure 4:**
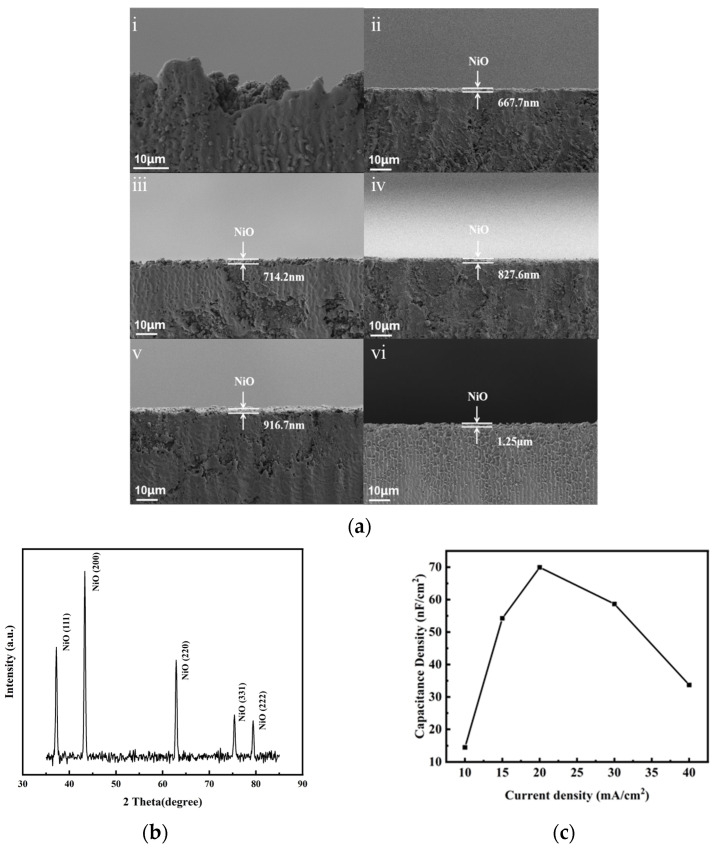

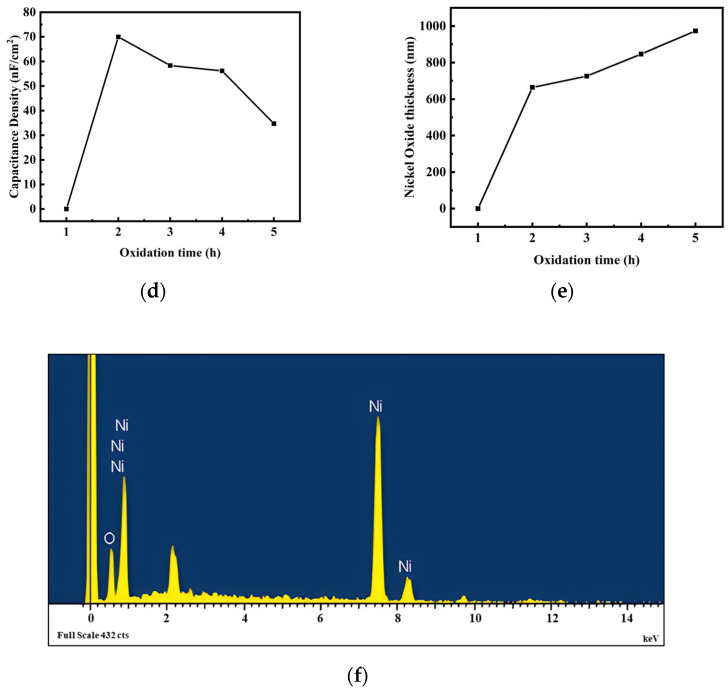
(**a**) SEM micrographs of porous NiO films under varied oxidation durations ((**i**–**vi**) correspond to the time period for high-temperature oxidation of 1 h, 2 h, 3 h, 4 h, 5 h, and 6 h); (**b**) XRD of the surface oxide layer after oxidation at high temperature; (**c**) capacitance density vs. current density; (**d**) dielectric thickness evolution versus holding time; (**e**) capacitance density dependence on oxidation time; and (**f**) EDS analysis results of the capacitor surface.

Figure 4c illustrates the current density dependence of capacitance density in MIM capacitors fabricated via high-temperature oxidation and electrode sputtering. Capacitance density increases proportionally with current density below 20 mA/cm^2^, reaching maximum performance at this threshold. Beyond 20 mA/cm^2^, elevated current densities inversely correlate with capacitance density, confirming the optimal anodization condition identified through structural characterization.

The current density governs the charge transfer flux during anodization, directly modulating electrochemical reaction kinetics. At lower current densities, moderated reaction rates allow sufficient ion diffusion to sustain spatially uniform oxidation, generating ordered nanoporous morphology as evidenced by SEM characterization. Elevated current densities enhance synchronized nickel oxidation and dissolution, favoring hierarchical pore formation through self-regulated field-assisted etching. However, exceeding critical thresholds induces concentration polarization, where nickel ion depletion at the electrode interface creates localized etching disparities. Concurrent oxygen evolution at high overpotentials generates interfacial gas bubbles that mechanically disrupt pore uniformity. Furthermore, geometric field enhancement at structural protrusions initiates self-accelerating etching cycles, ultimately degrading architectural regularity through runaway electrochemical dissolution.

Following current density optimization, dielectric layers were formed through thermal oxidation of porous nickel substrates at 600 °C with holding times ranging from 1 to 6 h. Cross-sectional SEM analysis of laser-sectioned samples revealed oxidation time-dependent thickness evolution in the NiO layers (Figure 4a), as confirmed by XRD analysis (Figure 4b). Capacitance density measurements demonstrated a performance maximum at intermediate oxidation durations (Figure 4d); Figure 4e demonstrates the variation in oxide layer thickness as a function of oxidation time; Figure 4f shows the EDS results of the NiO dielectric layer in the capacitor.

Electrical characterization revealed dielectric failure at 1 h oxidation, evidenced by ohmic conduction between electrodes that precluded capacitive behavior. Progressive thickness increases with extended holding times, followed by the inverse proportionality principle of parallel-plate capacitance, as confirmed by cross-sectional SEM measurements. The 2 h oxidation condition achieved optimal balance, providing sufficient dielectric integrity to prevent electrical shorts while minimizing layer thickness for maximal capacitance density. The porosity and oxidation time together set a fundamental trade-off in the Ni–NiO–Pt MIM capacitors. Longer thermal oxidation produces a thicker NiO layer, raising the dielectric path length and thereby increasing breakdown voltage (since in a uniform dielectric V_bd_ ≈ E_bd_ × d). However, the nanoporous nickel scaffold creates local thickness variations: pores or thin oxide walls can act as weak spots if the film is too thin. At the same time, capacitance decreases as C∝1/d. In our data, 1 h oxidation gave essentially no insulating NiO (electrical short), whereas 2 h oxidation yielded a uniformly grown oxide that prevented breakdown while still giving high capacitance. Further oxidation only modestly increased the breakdown voltage but caused a sharp drop in capacitance. In other words, beyond the optimal thickness, the capacitance per area collapses even though the voltage tolerance continues to improve. This reflects the well-known dielectric trade-off: excessive oxide growth (or porosity) can introduce cracks or low-permittivity regions, so that additional thickness yields diminishing capacitance density despite higher breakdown field.

### 3.2. Morphological Characterization and Mechanism Analysis: Glass-Based Three-Dimensional Capacitors

**Figure 5:**
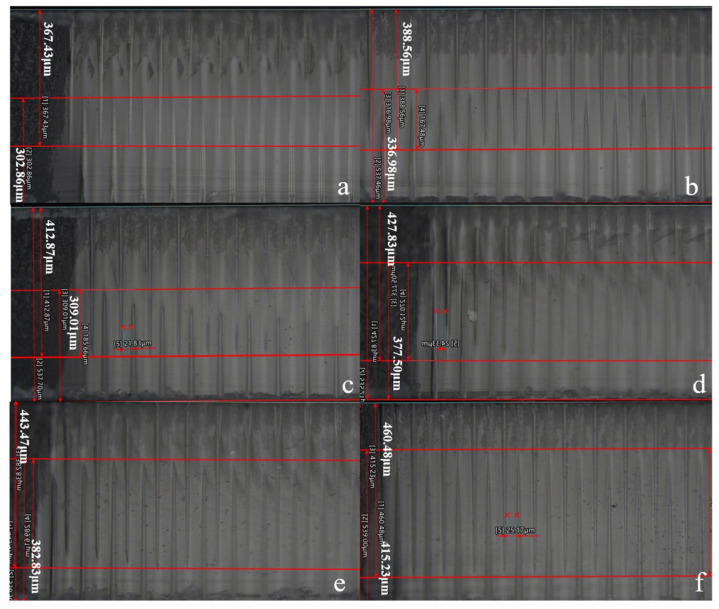
Cross-sectional images of interdigitated blind vias fabricated under varying laser parameters. Depth parameter: (**a**) 367 µm and 302 µm; (**b**) 388 µm and 336 µm; (**c**) 412 µm and 309 µm; (**d**) 427 µm and 377 µm; (**e**) 443 µm and 382 µm; (**f**) 460 µm and 415 µm.

Laser-induced etching enables the micromachining of glass dielectrics by coupling ultrashort-pulse laser irradiation with subsequent chemical etching. The underlying mechanism is laser-induced local modification: when a tightly focused laser beam is directed into the glass substrate, nonlinear optical effects trigger a nonthermal lattice transformation, producing a laser-affected zone (LAZ) that exhibits enhanced etchability. During the chemical etch step, the LAZ reacts with the etchant at a rate far exceeding that of the unmodified glass, allowing spatially selective removal of material and precise through-via formation. Prior to processing, the laser parameters were configured as follows: pulse duration of 1000 fs, two pulses per site, and average power of 12 W. Separate focal positions were set for the upper and lower blind vias: 3.65–3.19 mm for the upper vias and 3.16–3.60 mm for the lower vias. After HF etching, the resulting interdigitated structure is shown in Figure 5. The three-dimensional interdigitated geometry in Figure 5f is pronounced, with depths of approximately 460 µm and 415 µm for the upper and lower blind vias, respectively.

**Figure 6:**
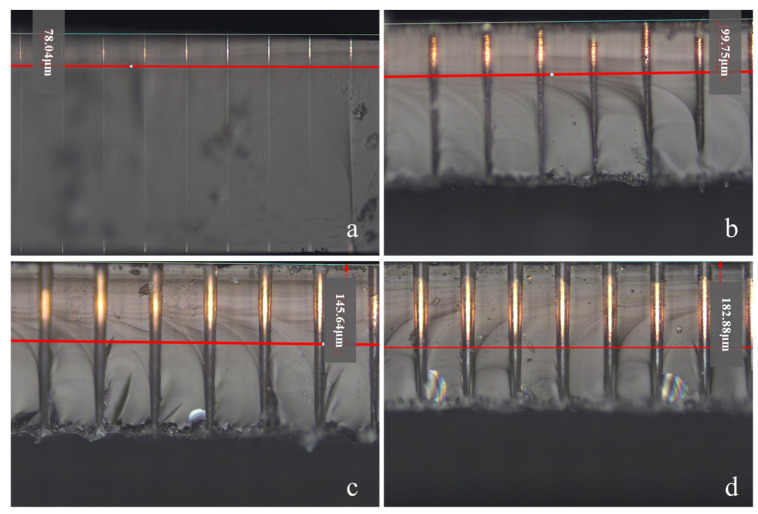
Metal filling of blind vias under deep-via sputtering conditions. Depth parameter: (**a**) 78.04 µm; (**b**) 99.75 µm; (**c**) 145.64 µm; (**d**) 182.88 µm.

Deep-via sputtering experiments were conducted on blind vias with varying diameters (10–40 µm), and their cross-sections were examined by optical microscopy. Copper was used as the sputtering target material, with the substrate maintained at 100 °C under an argon atmosphere at 0.5 Pa. The sputtering power and duration were set to 150 W and 30 min, respectively. Figure 6 shows the resulting cross-sectional micrographs. As the via diameter increased, the sputtered metal depth increased linearly; however, complete filling of the blind vias was not achieved at any diameter. The achievable aspect ratio (depth-to-diameter) for deep-via sputtering was approximately 5:1, which is insufficient to meet the high aspect-ratio requirements of three-dimensional interdigitated electrode capacitors.

**Figure 7:**
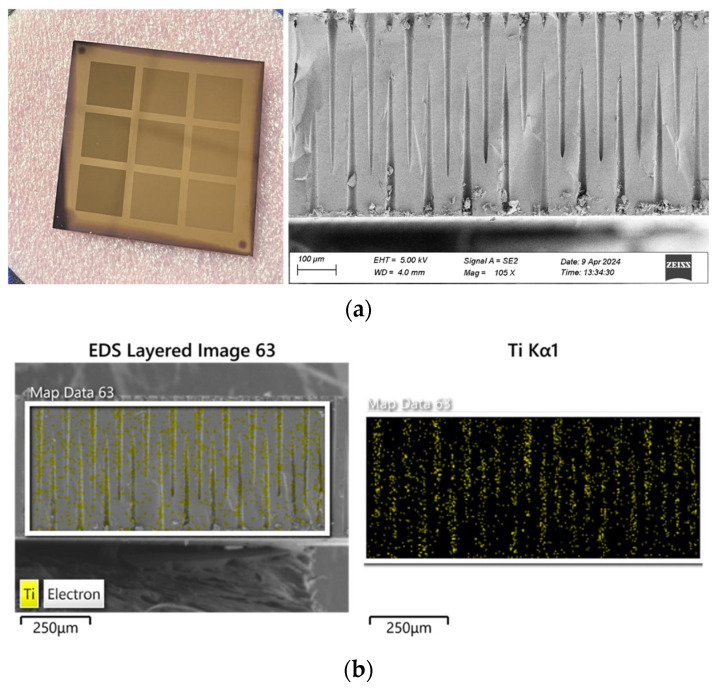
(**a**) Cross-sectional SEM image of ALD-filled metal; (**b**) EDS results.

Titanium nitride (TiN) thin films were deposited via atomic layer deposition (ALD) using TiCl_4_ and NH_3_ as precursors in alternating pulse sequences to achieve surface self-limiting reactions. Each deposition cycle comprised precursor exposure, inert-gas purge, reactant exposure, and a second purge, ensuring conformal film growth over three-dimensional features. The TiN electrode layers were prepared using a Beneq TFS-200 atomic layer deposition (ALD) system. The experimental temperature was controlled between 350 and 500 °C. TiCl_4_, which served as the liquid precursor, was maintained at 20 °C, with its flow rate regulated by both its volatility and the aperture of the gas valve. NH_3_ with a purity of 99.999% was used as the reactant gas. The flow rates of the two reaction gases were set between 200 and 2000 sccm. In a single ALD cycle, the pulse duration for TiCl_4_ was 150 ms, and that for NH_3_ was 100 ms. The chamber pressure was maintained at 500 Pa. A photograph and cross-sectional SEM image of the three-dimensional capacitor filled by ALD are shown in Figure 7a, and the corresponding energy-dispersive X-ray spectroscopy (EDS) elemental maps are presented in Figure 7b. The EDS scans reveal that the conductive TiN layer grown on the interdigitated blind-via surfaces is highly uniform. Owing to the intrinsic self-limiting surface chemistry of ALD, Ti atoms are deposited with atomic-scale precision onto the via sidewalls and bottoms, forming a continuous, dense metal fill. This conformal coverage markedly surpasses that achievable by conventional plating or sputtering, not only fully filling the blind vias but also ensuring uniform conductive-layer thickness between interdigitated electrodes.

### 3.3. Electrical Performance Testing of Porous Nickel Oxide MIM Capacitor

**Figure 8:**
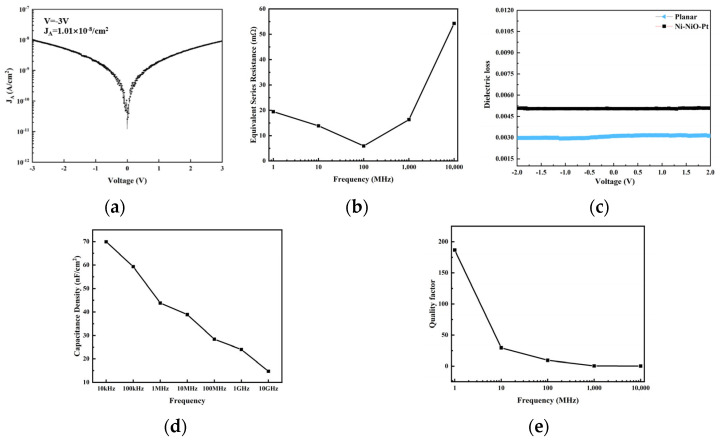
(**a**) Leakage current density testing; (**b**) ESR of the capacitor from 1 MHz to 10 GHz; (**c**) dielectric loss testing; (**d**) capacitance density over the frequency range from 10 kHz to 10 GHz; (**e**) quality factor for 3D porous NiO capacitor.

This work exhibits exceptional leakage current characteristics, demonstrating a leakage current density of 1 × 10^−^⁸ A/cm^2^ at 3 V. The 3D porous NiO capacitor demonstrated 14.8 nF/cm^2^ capacitance density at 10 GHz (Figure 8d). As the frequency increases, the quality factor experiences significant degradation (Figure 8e). The NiO capacitor shows pronounced Q-factor degradation at microwave frequencies; a practical device is modeled as an ideal C in series with a finite equivalent series resistance (ESR). The ESR captures losses from dielectric conduction, electrode resistance, and interface imperfections. Under AC excitation, the dissipation factor is DF = ωC·ESR (so Q = 1/(DF) = 1/(ωC·ESR)). Thus, Q falls rapidly as frequency ω increases or as ESR grows. In our structures, the porous Ni and thin Pt electrodes have nonzero sheet resistance (worsened by skin effect at GHz), and the NiO dielectrics have finite loss tangents; all of these effectively increase ESR at high frequency. Imperfect Ni/NiO/Pt interfaces could add localized loss channels as well. Rising series resistance and dielectric dissipation at GHz frequencies are responsible for the observed Q-factor drop—a conclusion consistent with standard RF capacitor models.

### 3.4. Electrical Performance Testing of Glass-Based Three-Dimensional Capacitors

**Figure 9:**
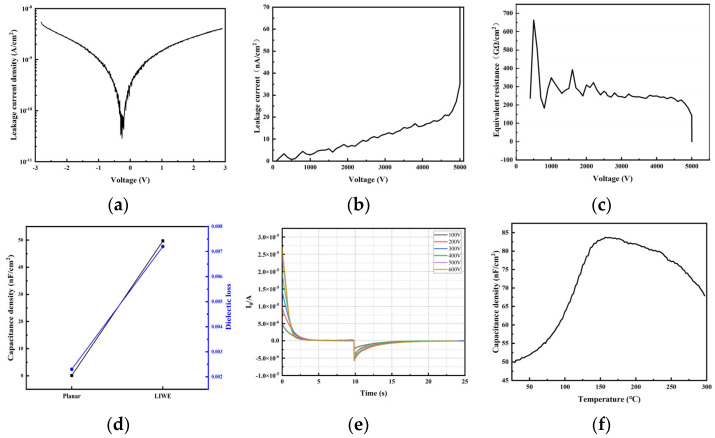
(**a**) Leakage current density testing; (**b**) leakage current versus electric field characteristics; (**c**) equivalent resistance versus electric field characteristics; (**d**) capacitance density and dielectric loss measurements; (**e**) cyclic charge–discharge characteristics at 100–600 V; and (**f**) effect of temperature on capacitance density.

In the glass-based three-dimensional capacitors, the use of ALD for electrode fabrication offers significant advantages in terms of uniformity and consistency. The measured leakage current density is as low as 5 × 10^−9^ A/cm^2^, as shown in Figure 9a. Benefiting from the low-temperature nature of the LIWE process, combined with sidewall roughness optimization methods, TGV (Through-Glass Via) arrays with exceptionally smooth sidewalls were achieved. High-voltage leakage current (Figure 9b) and equivalent resistance (Figure 9c) measurements demonstrate that the glass-based 3D capacitors exhibit a breakdown voltage up to 5000 V, with a breakdown field strength exceeding 8.2 MV/cm. Figure d presents the equivalent capacitance density and dielectric loss of the capacitors. Owing to the large increase in electrode surface area provided by the 3D interdigitated structure compared to a planar configuration, the equivalent capacitance density reaches 49.6 nF/cm^2^, representing an enhancement of approximately 439 times over planar glass capacitors. Figure 9e shows the high-voltage DC charge–discharge tests performed from 100 V to 600 V. From the curves, the energy conversion efficiency is calculated to reach up to 95%, indicating that the capacitors have the potential to play a critical role in high-power pulsed systems.

### 3.5. Discussion

Table 1 compares the key parameters of the 3D porous NiO capacitor with MIM capacitors employing various dielectric and electrode materials. In terms of comprehensive performance across these parameters, this work demonstrates the capability to meet application scenarios requiring specific parameter ranges. Compared to planar electrode-layer capacitors, it exhibits significant structural advantages and greater development potential. For example, high-permittivity materials can be deposited on its porous substrate to substantially enhance capacitance density.

Table 2 compares the performance of the glass-based 3D capacitors with several mainstream three-dimensional capacitor architectures. The glass-based device exhibits superior breakdown strength and energy efficiency, attributed to the high dielectric strength and low loss characteristics of glass, as well as the low electrode surface roughness achieved through the LIWE and ALD processes. These advantages indicate that glass-based 3D capacitors are particularly well-suited for high-voltage pulsed power systems.

## 4. Conclusions

In this research, the successful fabrication of two distinct 3D capacitors holds great significance. The Ni-NiO-Pt MIM capacitor with a 3D nanoporous structure offers a capacitance density 18 times higher than planar counterparts, enabling more energy storage in a small space, which is crucial for compact electronics and advanced sensors. Its fabrication process is cost-effective and BEOL-compatible, and it has excellent leakage performance while also serving as a platform for hybrid material systems and being environmentally friendly. The capacitor using TGV technology has a high aspect ratio interdigitated blind-via array. With glass’s outstanding insulating properties, it can withstand high voltages, making it suitable for high-power systems like high-voltage power supplies and pulse-generating equipment. Its low-loss property results in a 95% energy conversion efficiency, which is beneficial for energy-saving applications such as electric vehicle power management and renewable energy storage. Overall, these two capacitors drive technological progress in multiple fields and contribute to a more sustainable future.

## Figures and Tables

**Table 1:** Comparison of various MIM capacitors with different dielectrics and metal electrodes.

Reference	Insulator	Top Electrode	Capacitance Density	Current Density	Quality Factor
3D porous NiO capacitor This work	NiO	Pt	69.95 nF/cm^2^	1.01 × 10^−8^ A/cm^2^	186 (1 MHz)
T.H. Perng et al. [18]	HfO_2_	Cu	340 nF/cm^2^	5 × 10^−9^ A/cm^2^	-
D.H. Triyoso et al. [19]	Ta_2_O_5_	TiN	1590 nF/cm^2^	1 × 10^−5^ A/cm^2^	-
Chen, W.B. et al. [20]	PMNT	Pt	250 nF/cm^2^	1 × 10^−10^ A/cm^2^	17.4 (1 MHz)
Mariotti, C. et al. [21]	PVPh	Ag	3.3 nF/cm^2^	4 × 10^−9^ A/cm^2^	25 (1 GHz)
Cook, B.S. et al. [22]	PVP	Ag	2.2 nF/cm^2^	-	7.5 (3 GHz)

**Table 2:** Performance comparison between glass-based 3D capacitors and other 3D capacitor architectures.

Reference	Insulator	Capacitor Structures	Capacitance Density	Breakdown Strength	Energy Efficiency
3D Glass-based Capacitor This work	Glass	Interdigital electrodes	49.6 nF/cm^2^	8.2 MV/cm	95%
J. L. Li et al. [23]	NBT-0.45SBT	MLCCs	-	0.72 MV/cm	92%
F. M. Han et al. [24]	AAO	CNTs/AAO/CNTs	47 μF/cm^2^	8 MV/cm	-
P. Banerjee et al. [25]	Al_2_O_3_	TiN/Al_2_O_3_/TiN	10~100 μF/cm^2^	4.1 MV/cm	-
Q. B. Yuan et al. [26]	BaTiO_3_-Bi(Mg_0.5_Zr_0.5_)O_3_	Ceramics	-	3 MV/cm	89.7%

## Data Availability

Data are contained within the article.
